# The impact of household size on measles transmission: A long-term perspective

**DOI:** 10.1016/j.epidem.2024.100791

**Published:** 2024-09-06

**Authors:** Subekshya Bidari, Wan Yang

**Affiliations:** a Department of Epidemiology, Mailman School of Public Health, Columbia University, New York, NY, USA; b Herbert Irving Comprehensive Cancer Center, Columbia University Medical Center, New York, NY, USA

**Keywords:** Infectious diseases, Measles, Household transmission, Age structured transmission

## Abstract

Households play an important role in the transmission of infectious diseases due to the close contact therein. Previous modeling studies on disease transmission with household-level mixing have explored the relationship between household size distribution and epidemic characteristics such as final epidemic sizes and the basic reproduction number but have not considered the epidemic impact of declining household sizes caused by demographic shifts. Here, we use a disease transmission model that incorporates demographic changes in household sizes to study the long-term transmission dynamics of measles in communities with varying household size distributions. We explore the impact of incorporating both household- and age-structured mixing on the dynamic properties of the transmission model and compare these dynamics across different household size distributions. Our analysis, based on the household- and age-structured model, shows that communities with larger household sizes require higher vaccination thresholds and bear a greater burden of infections. However, simulations show the apparent impact of changing household sizes is the combined result of changing birth rates and household mixing, and that changing birth rates likely play a larger role than changes in household mixing in shaping measles transmission dynamics (*n.b*, life-long immunity makes replenishment of population susceptibility from births a crucial transmission driver for measles). In addition, simulations of endemic transmission of measles within a hypothetical population formulated using aggregated world demographic data suggest the decline in household size (driven by changing fertility rates of the population), in addition to increasing vaccination coverage, could have had a significant impact on the incidence of measles over time.

## 1. Introduction

Households play an important role in the transmission of infectious diseases. Contacts within households tend to be more frequent and last longer, leading to increased potential for transmission of infectious diseases. Consequently, numerous studies have examined the effect of household-level mixing in the spread of diseases (Becker and Hall, 1996; Becker and Starczak, 1997; House and Keeling, 2009). These studies have yielded valuable insights into the relationship between household size distribution and epidemic characteristics such as the basic reproduction number, final epidemic size, and optimal vaccination strategies (Becker and Dietz, 1995; Ball and Neal, 2002; Ball et al., 2009). However, the assumption of a static distribution of household sizes, often made in these studies, may not hold when studying endemic disease dynamics or accessing the long-term vaccination policy, particularly in populations undergoing significant demographic changes. Furthermore, these studies also do not consider the age structure in mixing, a critical factor in most childhood infections (Schenzle, 1984; Fine and Clarkson, 1982). To date, studies examining the effect of changing demographics and dynamic age and household structure on disease transmission are relatively scarce. While studies have developed individual-based models of household transmission (Glass et al., 2011; Geard et al., 2015) which offer flexibility in incorporating demographic changes, these models are often computationally expensive. In this study, we extend a model of household- and age-structured transmission introduced in Hilton and Keeling (2019) to study the long-term dynamics of measles in changing demographics using a system of ordinary differential equations (ODEs; referred to as the HH model hereafter). This compartmental modeling framework enables us to study the impact of household structures (particularly, household sizes and the changes over time) on transmission dynamics of the modeled diseases along with other key transmission drivers (e.g., age-structured mixing and vaccination), while avoiding the computational intractability of agent-based simulations.

Over the past century, populations in most nations have experienced substantial demographic changes, including increased life expectancy and reduced fertility rates, resulting in smaller household sizes and an aging population. These shifts have had a profound impact on population demographics, household structures, mixing patterns, and consequently the dynamics of childhood diseases such as measles. Mathematical models of measles transmission have used the SIR (susceptible–infectious–recovered) model framework with homogeneous population mixing (Earn et al., 2000; Bauch and Earn, 2003), or incorporated age structure to capture the heterogeneity in mixing between susceptible and infectious individuals (Schenzle, 1984; Bolker and Grenfell, 1993). These studies and mathematical modeling have been successful in replicating observed dynamics of measles through the interaction of non-linear dynamics, stochasticity, and seasonal forcing, particularly in the pre-vaccination times and when vaccination coverage is not very high. However, in the post-vaccination era when mass vaccination and demographic factors have become the primary drivers of measles transmission, more detailed models are likely needed to understand its persistent transmission (Measles reported cases, 2023). In this study, we apply the HH model with time-varying inputs informed by global demographics and vaccination data over the last five decades (1968–2019) to examine the impacts of demographic changes — particularly, how declining household size, as is becoming common in many parts of the world, may alter the dynamics of measles transmission in the context of mass vaccination.

Changes in household sizes are linked to birth rate (higher birth rates often imply larger number of children in a household and hence larger household sizes; here, the HH model determines the household size based on the fertility rate), and birth rate affects the replenishment of susceptible population and hence the transmission dynamics. As such, results from the HH model simulations represent the combined impacts of changing household sizes and birth rates. Nonetheless, we also conduct simulations controlling for susceptible birth rate to help tease apart the impact from birth rate changes. Overall, the HH model simulations elucidate the impacts of long-term demographic changes, as indicated by household sizes, on measles epidemic dynamics (e.g., periodicity), infection burdens, and vaccination threshold.

## 2. Methods

In this section, we describe the HH model and the simulation methods used throughout the paper. The model of measles transmission presented here is based on the *household infectious disease model* introduced by Hilton and Keeling (2019). The authors in Hilton and Keeling (2019) introduced a deterministic model that incorporates both age- and household structure into the dynamics of infectious disease, and compared the early growth and equilibrium behavior of the model for two different demographic scenarios. Here, we extend the model to capture both the evolution of household structures and dynamics of disease transmission to study the long-term dynamics of endemic diseases. In addition, we added an explicit vaccination component, a latent infection state, seasonality, and stochasticity to capture the stochastic transmission process crucial in the mass vaccination era. As introduced in Hilton and Keeling (2019), the HH model is a non-linear system of ODEs written in a matrix–vector form as below:

(1) $$\frac{dH}{dt}=H\left(Q_{Demo}(H)+Q_{Int}+Q_{Ext}(H)\right)$$

where H(t) is a vector of all possible (demographic and epidemiologic) state distribution at time t, $Q_{\text{Demo}}(H)$ is a matrix containing all demographic transition and vaccination rates,$Q_{\text{Int}}$ contains transmission rates for within household transmission, and $Q_{\text{Ext}}(H)$ contains age-structured external transmission rates.

We briefly outline the incorporation of demographic and epidemiological processes in the HH model in this section. A detailed description including the demographic and epidemiological process can be found in Hilton and Keeling (2019) and the accompanying supporting information.

### 2.1. Demographic model

**Fig. 1.**
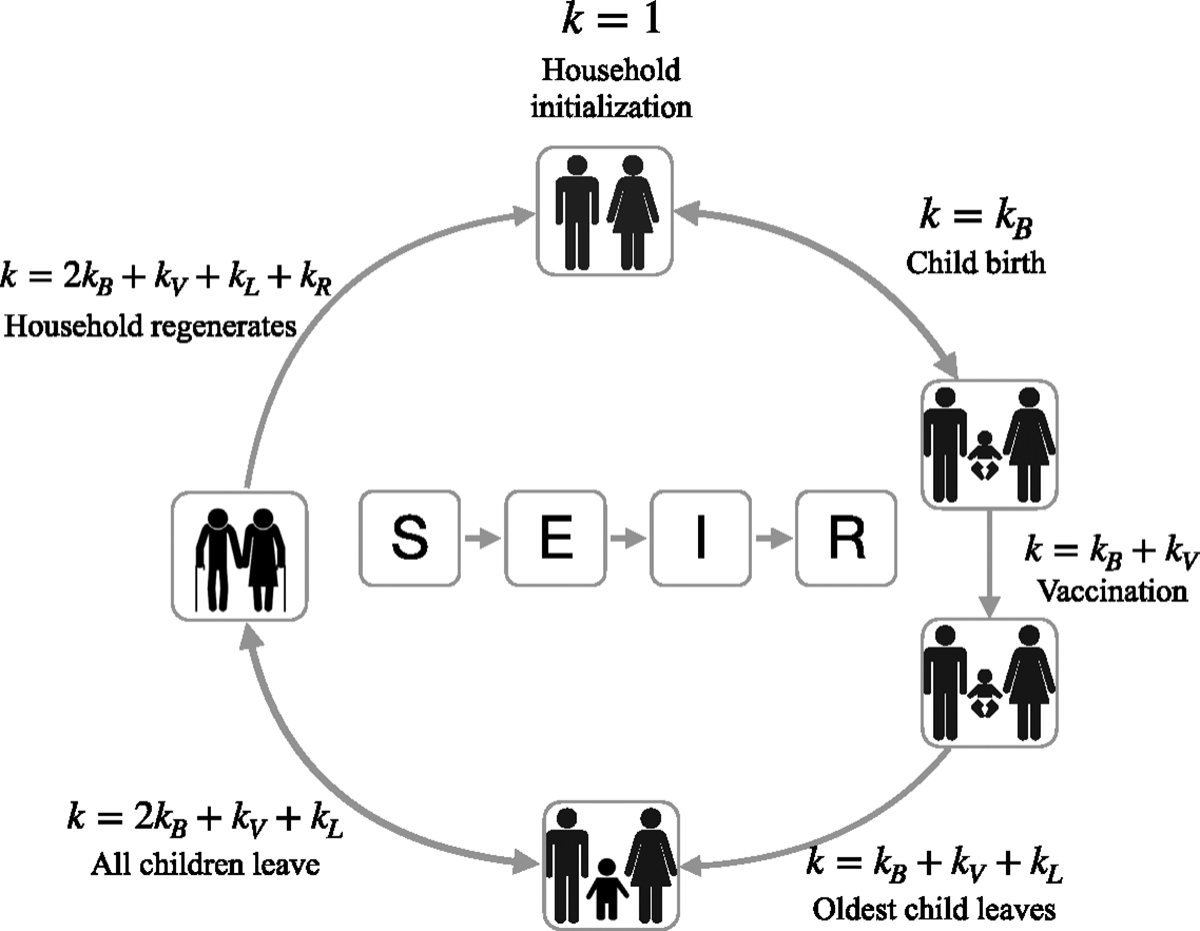
Household SEIR model. A simplified illustration of the demographic and epidemiological processes incorporated in the model.

As in Hilton and Keeling (2019), the demographic model describes the evolution of a nuclear family initialized with two individuals (parents). The parents can reproduce, adding individuals to the household. The size of a household increases when a child is born and decreases when an adult child leaves the parents’ house. The age of the adult children leaving home is determined using the average age at first marriage. When all the children leave, the parents remain in the household for some time dictated by life expectancy and are then replaced by a new couple whose immunological profile is that of the adult children leaving home (see Fig. 1 for an illustration of events in the demographic model).

In the HH model, the demographic state of a household is defined uniquely by the values of two parameters: the total number of individuals in the household N(=S+E+I+R) and the demographic counter k. Specific values of k trigger specific events: a household is initialized at k=1. At $k=k_{B}$, a child is born into the household, adding an extra susceptible individual to the household (note the model does not account for maternal immunity). For subsequent childbirth, the sequence $k=1,\ldots ,k_{B}$ is repeated for the number of children born which is informed by the family size distribution of the population. Thus, the counter k increases to $k_{B}+1$ only when the last child is born. At $k=k_{B}+k_{V}$, the youngest child is vaccinated. The eldest child leaves home at $k=k_{B}+k_{V}+k_{L}$, after which the sequence $k=k_{B}+k_{V}+k_{L}+1,\ldots ,\;2k_{B}+k_{V}+k_{L}$ is repeated for each subsequent adult child leaving. If the household has only one child, then the counter value is increased directly to $k=2k_{B}+k_{V}+k_{L}$ after the child leaves home. The counter values above $k=2k_{B}+k_{V}+k_{L}$ are associated with elderly couples. At the maximum counter value $k\;=2k_{B}+k_{V}+k_{L}+k_{R}$, the elderly couple is replaced by two new individuals. The k values can be mathematically interpreted as the shape parameters of Erlang distributions that define inter-event waiting periods. We set these values to be small ($k_{B}=k_{L}=k_{R}=2$, and $k_{V}=1$) to keep the total number of states from growing too large.

The rates of transitions in the demographic transition matrix $Q_{\text{Demo}}(H)$ are determined by the demographic parameters and the counter values, for instance, the rate of transition from k=1 to $k=k_{B}$ is $k_{B}/T_{B}$. To formulate the demographic parameters, we used the globally aggregated values of life expectancy, age at first marriage, and fertility rate from 1968 to 2019 (see Table 1 for a summary). The distribution of the number of children in a household is obtained by sampling from a binomial distribution with the mean equal to the average number of children in a household in a given year and the number of trials equal to the maximum allowable number of children (here taken as 10). The mean interval between birth and the age at vaccination is taken to be fixed values of 2 years and 1 year, respectively.

### 2.2. Epidemiological model

The modeled epidemiological dynamics follow a susceptible–exposed–infectious–recovered process with five events: (i) exposure inside the household, (ii) exposure outside the household, (iii) becoming infectious, (iv) recovery, and (v) vaccination. The transition events and rates associated with each are:

- Household exposure: $\left(S,E,I,R,k)\overset{\beta_{\text{int}}(t)\frac{SI}{N-1}}{\to }(S-1,E+1,I,R,k\right)$
- External exposure: $\left(S,E,I,R,k)\overset{\lambda_{\left(N,k\right)}(t)S}{\to }(S-1,E+1,I,R,k\right)$
- Becoming infectious: $\left(S,E,I,R,k)\overset{\sigma E}{\to }(S,E-1,I+1,R,k\right)$
- Recovery: $\left(S,E,I,R,k)\overset{\gamma I}{\to }(S,E,I-1,R+1,k\right)$
- Vaccination: $\left(S,E,I,R,k)\overset{\nu }{\to }(S-1,E,I,R+1,k\right)$

where S, E, I, and R are the number of susceptible, exposed, infectious, and removed (i.e., either recovered after infection or immune after vaccination) individuals in a household, respectively; N is the number of individuals in a household; k is a counter that indicates the demographic state of the household; $\gamma^{-1}$ and $\sigma^{-1}$ are the infectious and latent period respectively, in days; $\beta_{int}(t)$ is the internal transmission rate; $\lambda_{\left(N,k\right)}(t)$ is the age-structured force of infection responsible for transmission outside the household; and ν is the effective population vaccination rate, combining vaccination coverage with vaccine effectiveness (e.g., for a vaccination coverage of 60% and vaccine efficacy of 90%, the effective vaccination coverage rate v=60%×90%=54%).

The seasonal variation in measles transmission (e.g., due to the mixing of school children or climate conditions) is included in the model using a sinusoidal forcing term in both the household and external transmission as

(2) $$\beta_{\text{int}}(t)=\beta_{\text{int}}(1+\alpha \;cos(\frac{2\pi t}{365}))$$

(3) $$\lambda_{\left(N,k\right)}(t)=\lambda_{\left(N,k\right)}(1+\alpha \;cos(\frac{2\pi t}{365}))$$

where α is the amplitude of seasonal variation. Age-eligible individuals (i.e., 1 year-olds) are vaccinated according to the reported effective vaccination rates. In this study, to focus on the population-level impact of household sizes on measles transmission in the mass vaccination era, we did not specifically model additional heterogeneity due to clustering of vaccinations among family members (Kuylen et al., 2020).

The average internal (i.e., household) transmission rate is calculated as $\beta_{int}=\tau d_{int}$ where $d_{int}$ is the average time per day spent exposed to the members of the household and τ is the unit time rate of transmission of infection. Note that as detailed in Hilton and Keeling (2019), age structure is intrinsic to the HH model as different ages of the family members trigger demographic events that define household structure in the model. Further, variation in contact rates by age group (represented by age-structured mixing) is a key transmission factor for many infections including measles (Schenzle, 1984; Magpantay et al., 2019). The age-structured mixing is captured in the model component for the external transmission, which occurs through age-structured contacts and assumes each individual is additionally exposed to external infection outside the household based on the contact rate of their age group. In the simulations, we use 4 age groups (<1, 1–14, 15–50, and >50 years), and define the external transmission rate using a 4 × 4 external contact matrix D, where $D_{i,j}$ is the average time per day an individual in age class $C_{i}$ is exposed to members of age class $C_{j}$. Since the age of individuals in the household is not directly observed but is related to the demographic class (N,k), the age-structured force of infection $\lambda_{\left(N,k\right)}$ on individuals (in a household in demographic class (N,k)) is calculated by inferring a household age profile and applying the age-structured infection to this profile. The details of this calculation can be found in Hilton and Keeling (2019). To parameterize contact time within and outside households, we use the contact data provided by the POLYMOD study (Mossong et al., 2008).

### 2.3. Model simulation

To simulate the HH model, we first compute the disease-free equilibrium state for a given distribution of household sizes, as in Hilton and Keeling (2019). This disease-free equilibrium state $H_{I=0}$ is the eigenvector associated with the largest eigenvalue of the demographic transition matrix $Q_{\text{Demo}}(H)$. Then, we introduce one infected individual in a household with one child, and solve Eq. (1) numerically to obtain the vector summarizing the demographic and infectious state of the population at time t, denoted H(t). The population level infection prevalence is then given by

(4) $${\overset{‾}{I}}_{N,k}=\frac{\sum_{S,E,I,R,N,k}H_{S,E,I,R,N,k}I}{\sum_{S,E,I,R,N,k}H_{S,E,I,R,N,k}N(k)}.$$

Following the approach in Hilton and Keeling (2019), we use the early growth rate of infection, r, to compute the basic reproduction number, $R_{0}$ for the models. We compute the growth rate r based on the epidemic exponential growth period and calculate $R_{0}$ using the relationship between growth rate r and $R_{0}$ as given by the equation (Wallinga and Lipsitch, 2007):

(5) $$R_{0}=\left(1+\frac{r}{\gamma }\right)\left(1+\frac{r}{\sigma }\right).$$

To ensure comparability between different models, we scale the transmission rate τ such that $R_{0}$ for each model being compared is the same (see section S1 in supplement information). To determine how the inclusion of household mixing affects the dynamics of measles transmission, we use fixed values of infectious period $\gamma^{-1}=5$ days and latent period $\sigma^{-1}=8$ days, representative of measles. The values of basic reproduction number and amplitude of seasonality are taken to be $R_{0}=16$ and α=0.10 unless stated otherwise.

In Section 3.1, we begin with a comparison of the dynamics of the HH model for communities with differing household sizes. We simulate communities with different average household sizes (represented by the average number of children per household: ${\overset{‾}{N}}_{C}=2,\;3,\;\text{and}\;4$) and compare the equilibrium dynamic behavior of these models using tools from bifurcation theory (see supplementary information S2 for details). In Section 3.2, we examine how the average household size affects infection burden in the community using four representative household sizes (${\overset{‾}{N}}_{C}\;=\;1.5,\;2,\;3,\;\text{and}\;4$). To segregate the effect of household size and birth rate, we also perform simulations holding the rate of susceptible births constant. Specifically, for model populations with larger household sizes (${\overset{‾}{N}}_{C}=2,\;3,\;\text{and}\;4$), we vaccinate a fraction of newborns at the time of birth such that the rate of susceptible births entering the model population is the same as when the household size is ${\overset{‾}{N}}_{C}=1.5$. The infection burden here is defined as the cumulative number of infections per 100,000 people over a period of time (20 years here) after discarding the initial 100-year transient dynamics, across households of different sizes. In Section 3.3, we examine how the vaccination threshold for measles is influenced by household sizes. For the results presented in Sections 3.1–3.3, the model simulations are run deterministically, hence fractional values are possible and the infection prevalence is rarely exactly zero. As such, we use a threshold value of 10^−6^, instead of zero, to identify periods with no sustained transmission. The vaccination threshold is then identified as the minimum level of vaccination coverage needed to disrupt sustained transmission (i.e., no infection prevalence > 10^−6^ for more than 1 year), after discarding the initial 100-year transient dynamics.

In Section 3.4, we compare simulated measles incidence using global average demographic data from 1968 to 2019 with a hypothetical scenario under which the household sizes remained unchanged. To obtain the continuous change in demographic parameters, we initialize the HH model using the demographic parameter for the year 1968, discard the first initial 100 years, and then run the simulations by updating demographic parameter every year (see supplemental information S3, for details on simulations).

## 3. Results

### 3.1. Dynamics of measles transmission vary with the average household size of the community

**Fig. 2.**
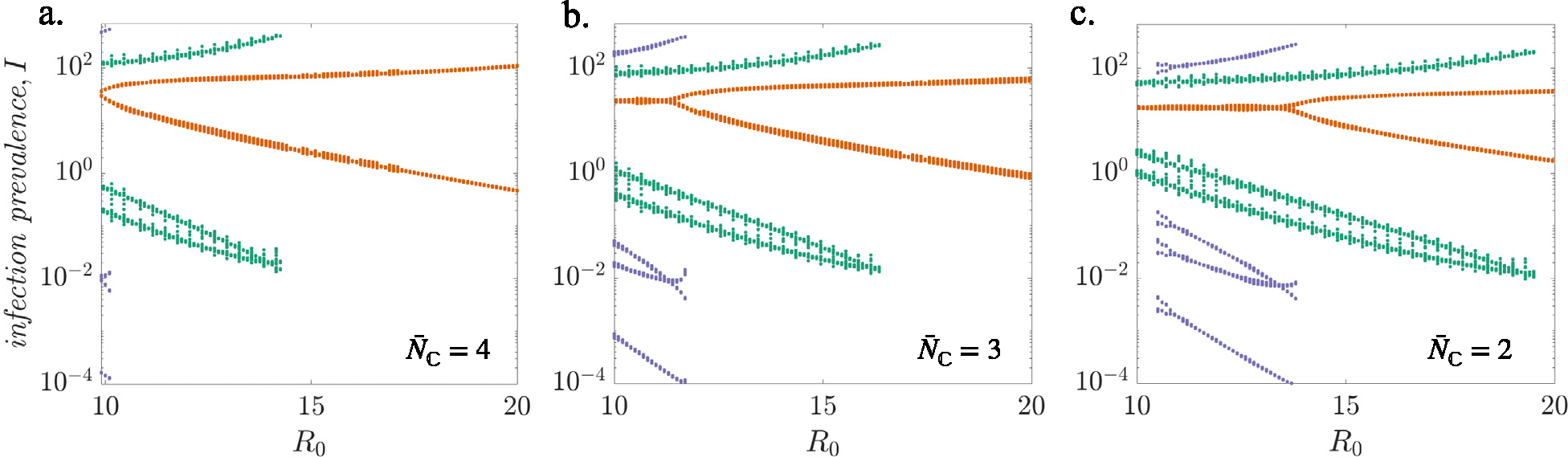
Comparison of model dynamics under different $R_{0}$ settings. Bifurcation diagrams show changes in epidemic dynamics as the basic reproduction number, $R_{0}$, is varied for different household sizes: (a) expected number of children ${\overset{‾}{N}}_{C}\;=\;4$, (b) expected number of children ${\overset{‾}{N}}_{C}\;=\;3$, and (c) expected number of children ${\overset{‾}{N}}_{C}\;=\;2$. Each dot represents log_10_(infectives) sampled annually on 90th day of the year (close to the peak) for 20 years after discarding a 100 years transient dynamics. Each attractor is identified with a different color. (For interpretation of the references to color in this figure legend, the reader is referred to the web version of this article.)

The qualitative behavior of the model varied based on the distribution of household sizes (Fig. 2a, b, and c). All household sizes considered have multiple attractors for lower values of $R_{0}$ with a dominant annual attractor that bifurcates to a biennial attractor as $R_{0}$ is increased (red curve in Fig. 2). However, the values of $R_{0}$ at bifurcations show a strong dependence on the household size of the community (Table 2). In particular, simulated epidemics undergo bifurcation at a lower value of $R_{0}$ in communities with larger household sizes than in those with smaller household sizes. For instance, the bifurcation points at which the annual attractor switches to a biennial attractor are $R_{0}^{*}=9.9$ for ${\overset{‾}{N}}_{C}=4$, $R_{0}^{*}=11.45$ for ${\overset{‾}{N}}_{C}=3$, and $R_{0}^{*}=13.6$ for ${\overset{‾}{N}}_{C}=2$. We find the epidemic dynamics in a community with larger household sizes resemble those in a community with smaller household sizes but under a higher $R_{0}$. This observation indicate that larger sized households have an inherently higher potential for disease transmission compared to their smaller sized counterparts.

For a value of $R_{0}=16$, typically associated with measles, a community with higher average household size ${\overset{‾}{N}}_{C}=4$ has a single biennial attractor (Fig. 2a) whereas communities with smaller household sizes, ${\overset{‾}{N}}_{C}=3$ and 2 have multiple attractors (biennial and triennial in Fig. 2b and c). The presence of multiple stable attractors in communities with smaller household sizes can lead to complicated dynamics due to shifts among the existing attractors and difficulty in prediction of outbreaks. Note that the increase in vaccination rate in the mass vaccination era could result in a similar effect. As the effective reproduction number is lowered by high vaccination coverage, less predictable outbreak cycles appear for all household sizes.

We also note that while the qualitative dynamics of the bifurcation diagram for the HH model is similar to the term-time forced SEIR model in Earn et al. (2000), the strong dependence of bifurcation points on the average household size stresses the need to consider the effect of household structure on transmission rates (also, see Supplemental Figure S3 for a bifurcation diagram corresponding to a wider range of $R_{0}$).

### 3.2. Simulated infection burdens vary by the average household size

**Fig. 3.**
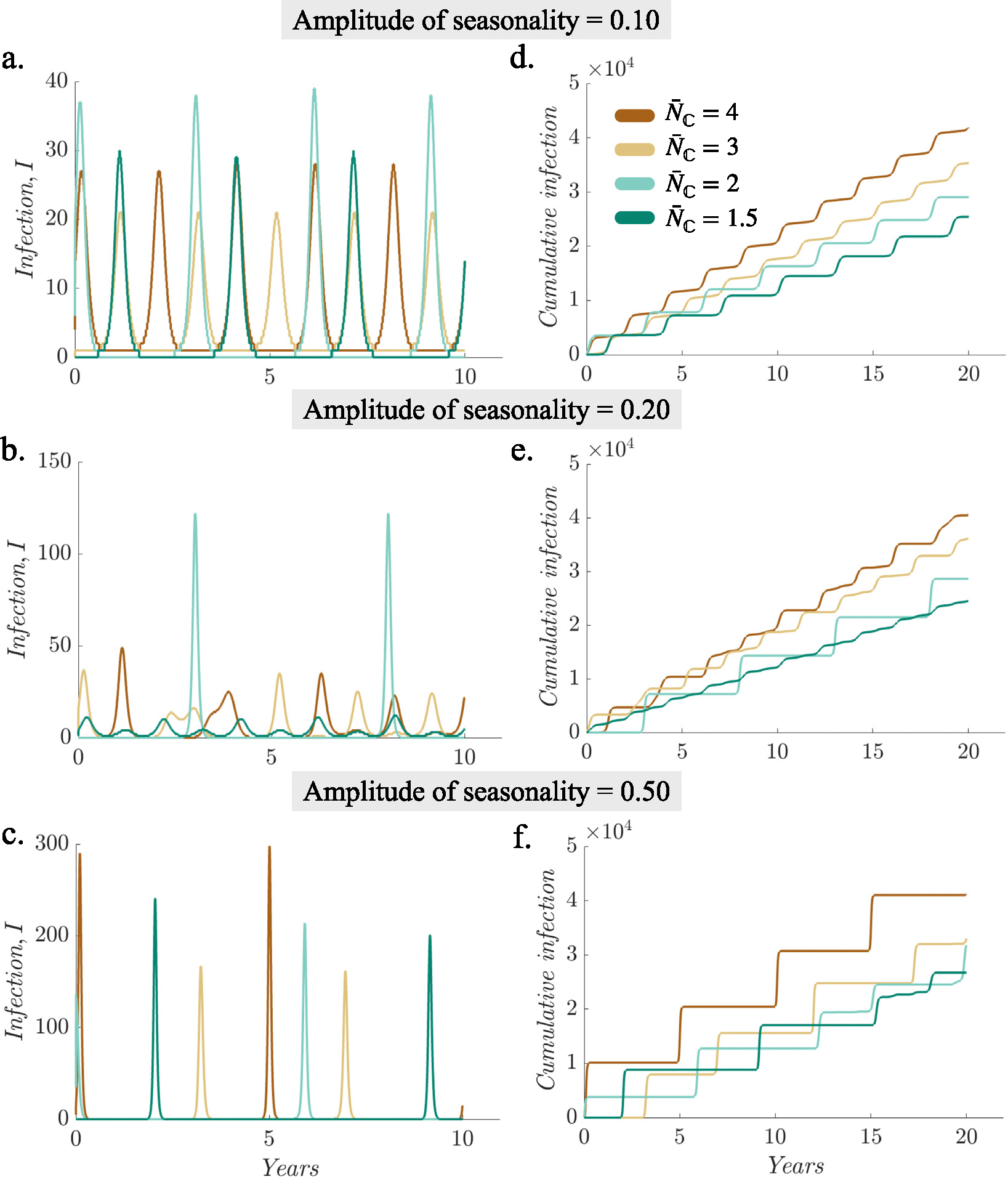
Simulated infection burden for different household sizes. The left panel depicts daily number of new infections per 100,000 individuals for different household sizes (see legend) and seasonal forcing: (a) amplitude of seasonality = 0.10, (b) amplitude of seasonality = 0.20, and (c) amplitude of seasonality = 0.50. The right panel (d, e, f) depicts corresponding cumulative infections for different household sizes and seasonal forcing.

The average household size and the seasonality parameter significantly influence epidemic dynamics. As the strength of the seasonality parameter is increased, epidemic dynamics and its relationship with household size also change. For instance, when α=0.10 (Fig. 3a), communities with larger household sizes (${\overset{‾}{N}}_{C}=4,\;{\overset{‾}{N}}_{C}=3$) experience biennial outbreaks and those with smaller household sizes (${\overset{‾}{N}}_{C}=2,\;{\overset{‾}{N}}_{C}=1.5$) experience triennial outbreaks; when α=0.20 (Fig. 3b), communities of all household sizes except ${\overset{‾}{N}}_{C}=2$ experience chaotic outbreaks; when the amplitude of seasonality was higher α=0.50 (Fig. 3c), communities of all household sizes experience outbreaks with large periods. Hence, to ensure a fair comparison between settings with different household sizes and infection dynamics, we compare the infection burden over a 20-year endemic period for settings with different household sizes (see Methods).

Model simulations show that communities with larger average household sizes sustain a greater per capita infection burden over time, even when other epidemic conditions such as $R_{0}$ and vaccination levels are held constant (compare the cumulative infections over the 20-year period: $I_{{\overset{‾}{N}}_{C}=4}=41867$, $I_{{\overset{‾}{N}}_{C}=3}=35333$, $I_{{\overset{‾}{N}}_{C}=2}=29048$, $I_{{\overset{‾}{N}}_{C}=1.5}=25415$ in Fig. 3d). Communities with larger average household sizes exhibit a greater overall burden of infection, as indicated by the larger infection tallies (Fig. 3d, e, and f).

**Fig. 4.**
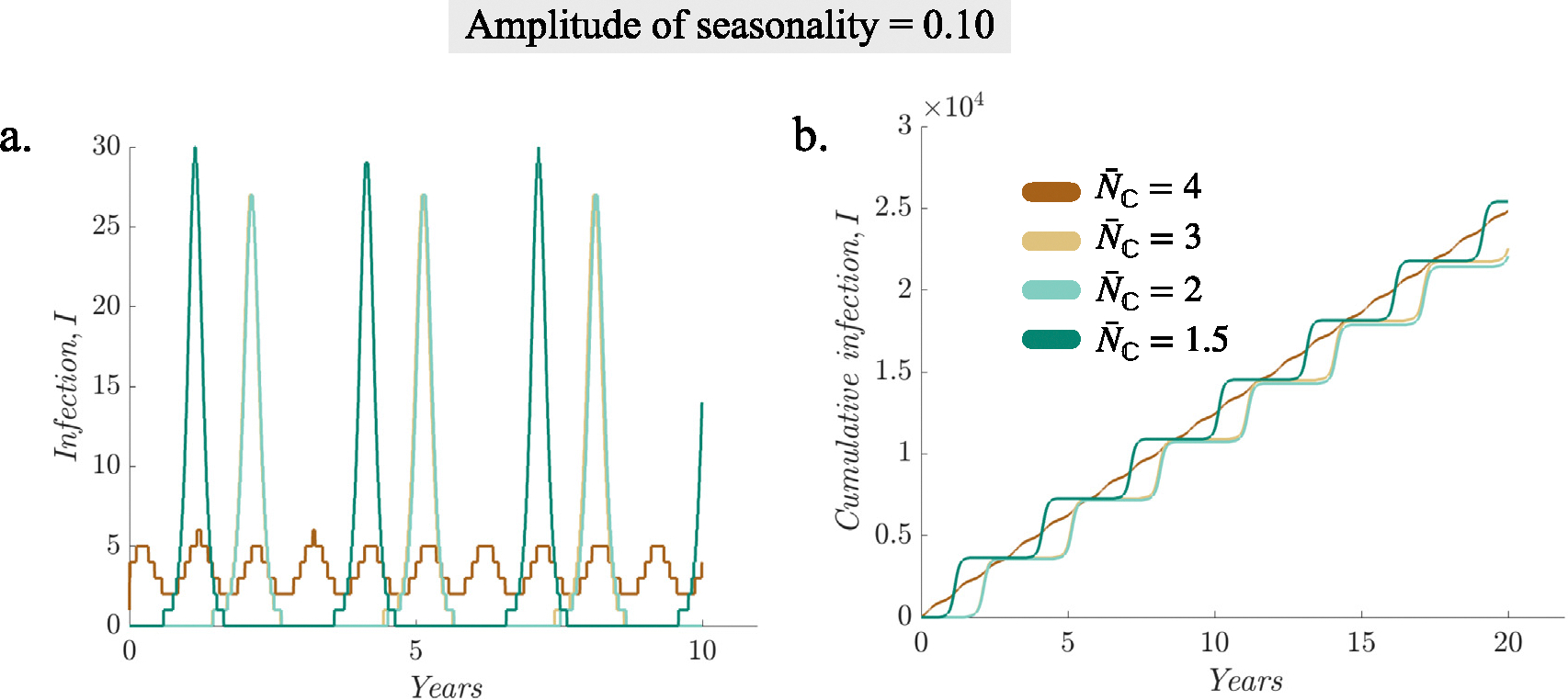
Simulated infection burden for different household sizes as the susceptible birth-rate is fixed. (a) Daily new infections per 100,000 individuals for different household sizes (see legend), (b) corresponding cumulative infections.

As noted above, the average household size is inherently linked with the population birth rate and configured as such in the HH model (the average household size is determined by the average number of children born in a family plus parents). To tease apart the effect of household size and birth rate, we compare infection burden across different household sizes as the rate of susceptible births is controlled (Fig. 4). Fig. 4 suggests that most of the differences seen in the infection burden across household sizes come from changes in birth rate. Nonetheless, nuanced differences remain after controlling for birth rate. In particular, communities with larger household sizes experience more persistent transmission (see brown and yellow curves showing annual outbreaks for ${\overset{‾}{N}}_{C}=3$ and 4 vs. green lines with less frequent outbreaks for ${\overset{‾}{N}}_{C}=1.5$ and 2 in Fig. 4), which is consistent with the higher transmission potential for larger sized households observed in Section 3.1.

### 3.3. Vaccination coverage required to prevent outbreaks varies by household size

An important goal of public health research and epidemiological modeling is to determine the vaccination coverage required to cease transmission of infections, commonly referred to as the vaccination threshold. Here we examine how the vaccination threshold for measles is influenced by household sizes. To determine the vaccination threshold, we run model simulations with different combinations of household size distributions and vaccination rates and observe when endemic infection ceases.

**Fig. 5.**
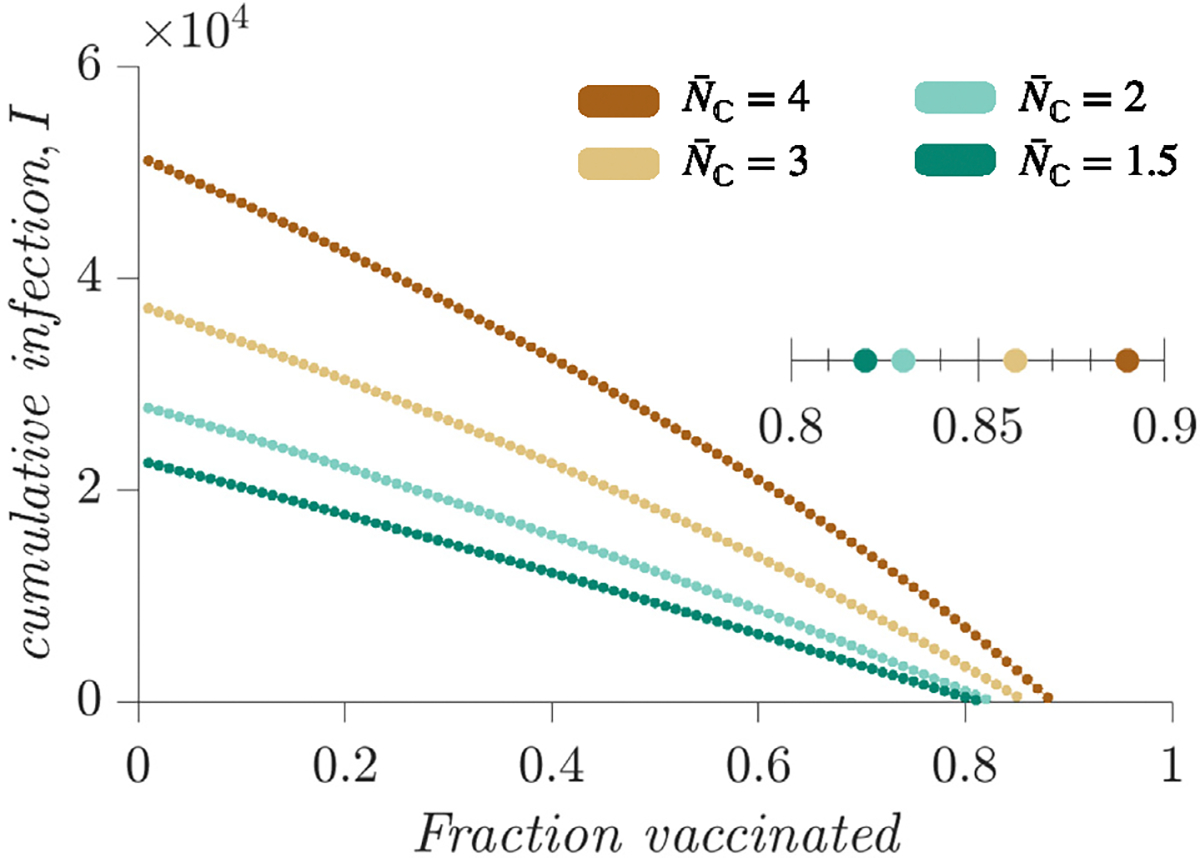
Vaccination threshold for different household sizes. Colored dots show total infection (per 100,000) over a 20-year period for each vaccination level (see x-axis). The estimated vaccination threshold for each average household size/number of children per household (see colored dots and legend) is shown in the inset. (For interpretation of the references to color in this figure legend, the reader is referred to the web version of this article.)

The simulation results demonstrate a correlation between the vaccination threshold and the average household size in the community (Fig. 5). We find that higher vaccination coverage is required to break transmission when the average household size is larger. For example, using model settings shown in Fig. 5, when the average household size is 3.5 (i.e., 2 parents +1.5 children on average), the estimated vaccination threshold is 82%; in contrast, when the average household size is 6 (i.e., 2 parents +4 children on average), the vaccination threshold increases to 89%. Based on results reported in Section 3.2 and Fig. 4, this correlation between household size and vaccination threshold likely comes from higher birth rate associated with higher average household size in the HH model.

### 3.4. Effect of demographic changes on measles incidence

**Fig. 6.**
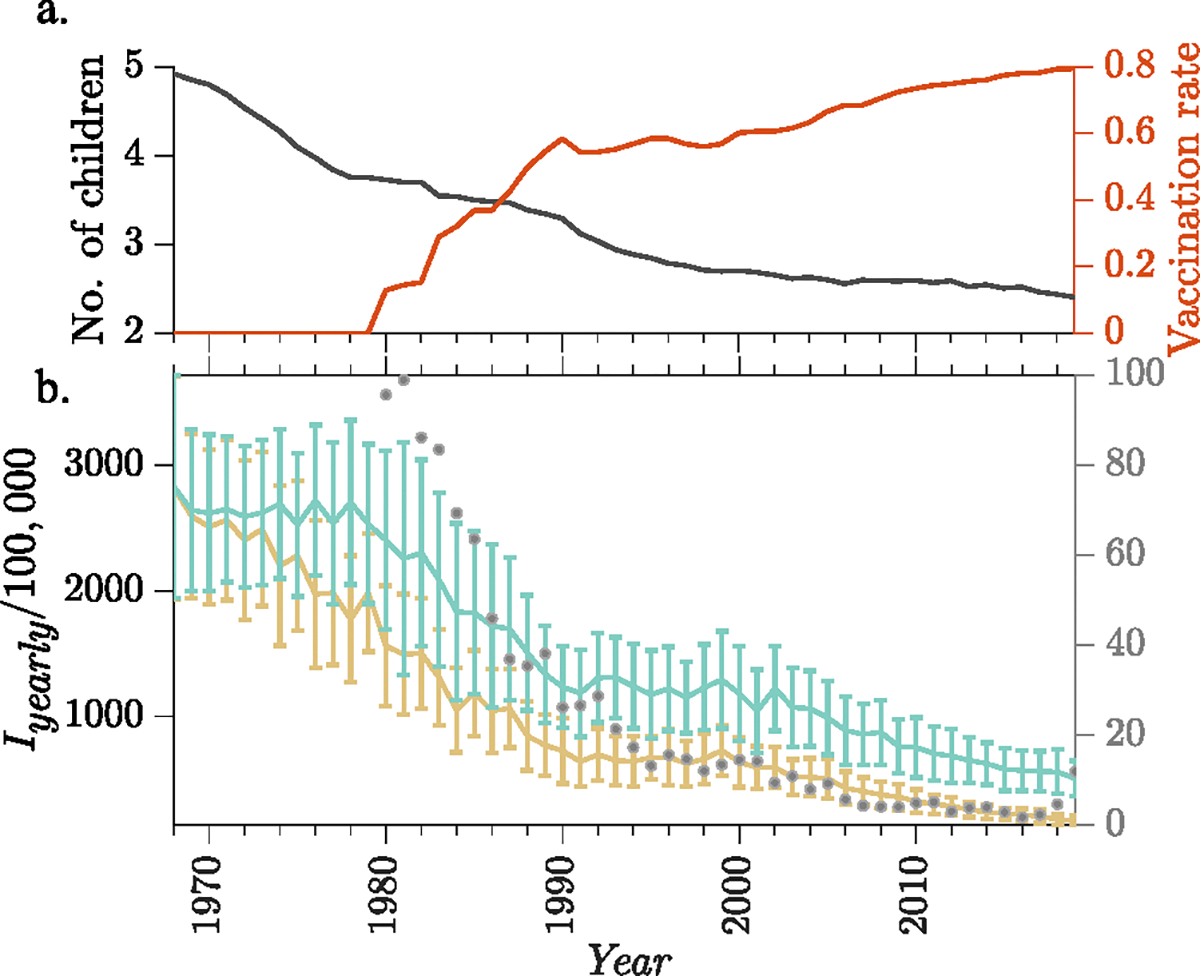
Effect of demographic changes on measles epidemic dynamics. (a) Average number of children per household (black curve, left y-axis) and effective vaccination coverage against measles (red curve, right y-axis) using globally aggregated data from 1968 to 2019. (b) Annual measles incidence (per 100,000 individuals) in the hypothetical population. The yellow curve shows mean annual measles incidence with yearly change in household sizes per the demographic data, and the green curve shows mean annual incidence under the assumption of constant household size. The error bars represent the standard deviations of the simulations which we run 20 times for each seasonality parameter values α={0.05,0.1,0.15,0.2}. The gray dots (right hand side y-axis) show available global measles incidence data reported by the World Health Organization (1980 and onwards). (For interpretation of the references to color in this figure legend, the reader is referred to the web version of this article.)

The dynamics of measles transmission is strongly correlated with the changes in demographics (e.g., birth rate and mixing patterns) and vaccination (Merler and Ajelli, 2014). Here, we examine the effect of declining household sizes on measles epidemic incidence. Incorporating yearly changes in demographic parameters allows us to model the endemic dynamics of measles more realistically. We specifically incorporate changes in household sizes into the model, a variable that is often assumed to remain constant in most other models. To access the impact of this inclusion, we compare the yearly measles incidence from 1968 to 2019, a period when the average global household size decreased from around 7 (2 parents + 5 children) in 1968 to around 4 in 2019 (Fig. 6a left *y*-axis show changes in household size; Fig. 6b yellow curve show annual incidence rates). We contrast this with yearly incidences in a scenario where the average household size remained unchanged (Fig. 6b green curve). In addition, both scenarios include changes in vaccine coverage against measles which started to increase globally around 1979 and plateaued at around 80% in 2019 (see Fig. 6a right y-axis).

Model simulations suggest that the increase in vaccination is primarily responsible for the decline in measles incidence during the post-vaccination era as indicated by the sharp decline in incidence from 1979 to 1990 corresponding to the period when vaccination was introduced and coverage increased rapidly. However, the decline in household sizes (driven by decreasing fertility rate) also appeared to play an important role in accelerating the reduction in measles incidence, as the green curve (no changes in household size) shows significantly higher incidence than the yellow curve (with the reported household size changes). For example, if the average household size remained around 7 during 1968–2019, the model predicted a mean yearly measles incidence of 1522 (±762; standard deviation) infections per 100,000 (green curve), whereas if the average household size decreased to 4.4, the predicted mean yearly incidence is 1028 (±791) infections per 100,000 (yellow curve). We note, again, this result largely reflects the impact of reduced replenishment of susceptible population due to reduced birth rate, as shown in Fig. 4.

## 4. Discussion

Mixing within households plays an important role in the transmission of infectious diseases, as the majority of individuals reside in households and tend to interact more closely with household members than outside contacts. To capture these important interactions and differential mixing patterns resulting from changes in household sizes over time, we compare endemic measles outbreaks in communities with different household sizes. Our results illustrate how infection dynamics, infection burden within the community, and the vaccination threshold required to break the chain of transmission vary by the community’s average household size, driven by changes in fertility rates. Furthermore, we applied the extended HH model to investigate the long-term dynamics of measles in a population undergoing demographic changes.

The changes in household sizes are inherently linked to the changes in birth rates in both real-world settings and in the HH model, making it challenging to disentangle the effects of household size alone from those of varying birth rates. For measles, our simulations controlling for birth rate (Fig. 4) suggest the simulated population-level impacts using the HH model largely come from changes in birth rate, with more nuanced differences by household size. This finding is likely a result of the life-long immunity of measles infection and vaccination, making susceptibility replenishment via births (i.e., birth rate) a main limiting factor of the disease system. Consistently, using an agent-based household model with dynamic births, deaths, and movements between households, Glass et al. (2011) concluded that ‘‘when immunity is assumed to be life-long, the pattern of births by household size is the key driver of infection’’. Merler and Ajelli (2014) also found that patterns in birth rates and vaccination are the main drivers of measles incidence using a non-stationary, age-structured disease transmission model. Importantly, our results still show that household size is a good indicator of changing population demographics (including the changes in birth rate). Thus, findings from this study reflect the overall impact of changing population demographics as indicated by the average household size, and we focus on discussing such overall impact below.

Our findings highlight the significance of changing population demographics (as indicated by household size) on measles dynamics and the importance of considering it in epidemiological models. Notably, the qualitative dynamics of the model varied significantly between communities with different household sizes. For the basic reproduction number corresponding to measles, we observed the emergence of multiple attractors for communities with smaller household sizes, whereas communities with larger household sizes exhibited a single biennial attractor. This suggests that the demographic shift towards smaller household sizes (e.g., driven by decreasing birth rate) can cause the dynamics of measles to switch from regular biennial cycles to more complicated dynamics with higher period cycles intermixed with annual/biennial cycles. While we cannot test this against empirical data from measles outbreaks as the vaccination coverage also increased in concert with the decline in household size, this observation could offer a possible explanation for the difference in measles epidemic dynamics between different regions in the pre-vaccination period. For instance, the difference in pre-vaccination measles dynamics such as regular biennial measles outbreaks in England and mixtures of annual, biennial, and triennial cycles in New York City with lower predictability (Grenfell et al., 1994) could be in part due to the different population demographics/household structures in the two regions.

The eradication of measles remains an ongoing challenge in many parts of the world. Several previous studies have examined the relationship between the vaccination threshold required to break the chain of transmission and household size (Becker and Dietz, 1995; Becker and Hall, 1996; Glass et al., 2011). The studies in Becker and Dietz (1995), Becker and Hall (1996) were theoretical analyses and included strong assumptions such as high transmission rate between members of the household (e.g., an infected individual in the household implied everyone in the household is infected), static household distribution, and a predetermined household size distribution or a fixed household size throughout the community. The study in Glass et al. (2011) used an agent-based dynamic household model to examine patterns of infection and immunity across populations with different household sizes and different fertility settings. Despite the different methodologies and modeling settings, these previous studies (Becker and Dietz, 1995; Becker and Hall, 1996; Glass et al., 2011) and ours consistently show the vaccination threshold increases with the average household size of the population. In addition, consistent with Glass et al. (2011), we find the infection burden increases with the average household size of the population. Together, these findings echo previous work (Ferrari et al., 2013; Conlan and Grenfell, 2007) emphasizing the role of local demographics in measles transmission and the need for elimination strategies tailored to local demographic conditions.

We demonstrate the importance of including changing population demographics (particularly changes in household size and birth rate) in modeling endemic disease dynamics through simulations of measles in a hypothetical population based on aggregated demographic data across all countries. Undoubtedly, these aggregated demographic data/parameters are not a good representation of the heterogeneity between different regions and do not fully capture the complexity in measles transmission across different regions. Our goal here was to highlight the differences in model outcomes as the population demographics (here, indicated by household sizes) vary, rather than precisely model global measles dynamics. Indeed, model-generated yearly measles incidence captures the general time trend as per data reported by the World Health Organization (Fig. 6b), despite being larger in magnitude (note we did not account for reporting rates, which likely have varied over time and regions). Future work in this direction could involve using regional surveillance data to calibrate these models and infer and compare key parameters across different regions. This approach could provide insights into differences in measles transmission between regions and inform eradication plans.

We note that we made some simplifying assumptions that may deviate from real-world observations. The model, assuming that households are comprised of nuclear families with parents and unmarried children, is a simplified representation of a much more complex process including single-person households, multi-generational households, or households with divorced parents. For instance, a study that analyzed data from various sources showed that 17.11% of the households in the United States and 20.26% of households in Japan were single-person households in 1970 with the numbers gradually increasing for most countries (Ortiz-Ospina and Roser, 2020). The model presented here does not include migration which has been suggested to be a key factor for the persistence and reemergence of measles in highly vaccinated populations (Yang et al., 2017, 2019). In future work, the transmission process described here can be modified to include migratory behavior to study measles spread and persistence in populations with high vaccination coverage.

Our study shows the apparent impact of changing household sizes from the HH model is the combined result of changing birth rates and household mixing, and that changing birth rates likely plays a larger role than changes in household mixing in shaping measles transmission dynamics. The latter finding may be largely due to the life-long immunity assumed for measles, which makes replenishment of population susceptibility from births a crucial transmission driver for the disease. Thus, the relative importance of household mixing may vary by disease depending on immune response and may be more pronounced for diseases without life-long immunity (given the less limiting impact of susceptibility from births) than shown here for measles. Overall, our simulations show changes in household size can be used as an indicator of population demographics, and the inclusion of key population demographics and interactions in epidemiological models can help inform the underlying transmission dynamics and public health interventions.

## Supplementary Material

1

## Figures and Tables

**Table 1:** Summary of parameters used in the model.

Parameter	Description	Values

$T_{B}$	Between birth interval	2 years
$T_{V}$	Vaccination age	1 year
$T_{L}$	Age at leaving parents’ house	22 years–28 years (World Marriage Data, 2019)
$T_{D}$	Life Expectancy	56 years– 73 years (World Population Prospect, 2022)
${\overset{‾}{N}}_{C}$	Average number of children in a household	5–2.4 (World Population Prospect, 2022)
v	Vaccination coverage rate	0–79.45 (Measles reported cases, 2023)

**Table 2:** Summary of the bifurcation points for the dynamic transition in the HH model as the basic reproduction number, $R_{0}$ is varied.

Household size	${\overset{‾}{N}}_{C}=4$	${\overset{‾}{N}}_{C}=3$	${\overset{‾}{N}}_{C}=2$

Annual	<9.8	<11.45	<13.6
Biennial	[9.9, 20]	>11.45	>13.6
Triennial	<14.25	<16.35	[7.7, 19.5]
Quadrennial	<10.1	<11	[10.5, 13.8]

## Data Availability

The data used in the study is freely available and the code is available at https://github.com/sbidari/HHmodel.available in github.
