# Supplementary material for: The impact of household size on measles transmission: A long-term perspective
Source: Epidemics. Author manuscript; Available in PMC 2025 Jan 16. (PMC11737883; doi:10.1016/j.epidem.2024.100791)
Supplement: 1 [file NIHMS2042796-supplement-1.pdf]

Supporting Information
The Impact of Household Structure on Measles Transmission: A Long-term Perspective

Subekshya Bidari, Wan Yang

S1 Relationship between basic reproduction number, R_0 and unit time transmission rate, τ

To facilitate comparison between models with different household sizes, we calibrate models with different household size to have the same value of R_0. This is achieved by scaling the unit time transmission rate, τ, to obtain the desired value of R_0 across all household sizes considered.

Using numerical simulations, we find that R_0 increases approximately linearly with τ for the range of values of τ used here. Thus, the relationship between R_0 and τ for a specific household size can be linearly approximated by a straight line (with $R^2 \geq 0.99$), Figure S1.

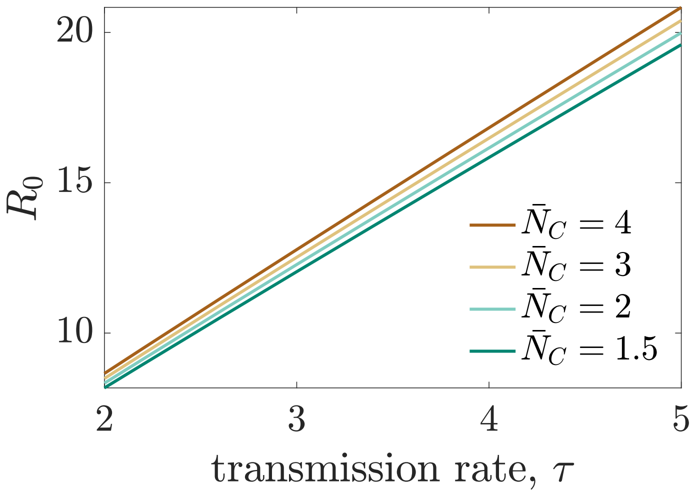

Figure S1: R_0 **increases linearly with transmission rate,** τ.

S2 Bifurcation analysis

The bifurcation diagrams shown in Fig 2 is constructed using a two-step process. In the first step, we construct a diagram using a fixed initial condition for all parameter values. Here, we used an initial condition of one infected individual in a household with one child. We numerically integrate the system of equations in Eq 1 while varying the parameter of interest (here R_0 and α). After integrating the system of equations over an initial period of 100 years to allow the dynamics to reach their long-term equilibrium state, we plot the population level infection prevalence (calculated using Eq 4) at a particular time-point each year (90th day of the year). This results in a bifurcation diagram where outbreaks with annual cycles are represented by a single dot, biennial cycles appear as two dots, and so on. However, when multiple attractors are present, the basins of attraction (region in the parameter space that maps to an attractor) can be highly irregular. Thus, the use of same

initial conditions for adjacent parameter values can lead to sudden jumps between attractors due to the changing shape of the basins of attractions of the multiple attractors. To avoid these sudden jumps, we use a continuation method to create bifurcation diagrams in the second step.

Under the continuation method, the simulations are run sequentially and the state of the system at the end of one simulation serves as the initial conditions for the next simulation - rather than all simulations using the same initial conditions. We identify the different attractors of the system based on the initial bifurcation diagram created in the first step. We then trace each attractor using the continuation method to create a new bifurcation diagram. This approach ensures that the changes in the bifurcation diagram come from the loss of stability of one attractor giving way to a new stable attractor as the control parameter is varied, rather than due to the initial conditions starting in different basins of attraction. Since multiple attractors can coexist for the same parameters depending on the initial conditions, the generation of a complete bifurcation diagram that identifies all the attractors of the system is not always possible. We show the bifurcations of the attractors observed in the initial bifurcation diagram and note that there might exist one or more attractor(s) of the system that was not observed in the initial bifurcation diagram.

S3 Stochastic simulations

Here, we describe the implementation of the stochastic version of the model presented in Equation (1) in the main text. Our implementation is based on the framework in [1]. This approach relied on recasting the system of differential equations as difference equations, where each class of households holds a whole number (vs. a fraction of all possible classes in the deterministic version) such that

$$H_{(i,t+1)} = H_{(i,t)} + H^{\text{in}}_{(i,t)} - H^{\text{out}}_{(i,t)} \tag{S1}$$

where $H^{\text{in/out}}_{(i,t)}$ is the number of households transitioning into/out of the class i at time step t and determined using the transition rate matrix $Q = Q_{\text{Demo}} + Q_{\text{Int}} + Q_{\text{Ext}}$ as described in [1]. We briefly summarize the process below:

1. For each class i at time t, the probability of any event occurring in related households during the time step is calculated as

   $$p^{\text{any}}_{(i,t)} = 1 - \exp\big(-\Big(\sum_{j \neq i} q_{ij}\Big)\delta t\big) = 1 - \exp\big(q_{ii}\delta t\big)$$

   where q_{ij} is the ij^{th} entry of the transition rate matrix Q.

2. For each class i at time t, we sample the number of events occurring from a binomial distribution with probability computed in step 1,

   $$H^{\text{any}}_{(i,t)} \sim \text{binomial}\big(H_{(i,t)}, p^{\text{any}}_{(i,t)}\big).$$

3. We then compute the number of households undergoing each event $H^{\text{event}}_{(i,t)}$ by sampling from a multinomial distribution

   $$H^{\text{event}}_{(i,t)} \sim \text{multinomial}(H^{\text{any}}_{(i,t)}, \mathcal{P}_i)$$

   where P is a matrix with elements p_{ij} such that

   $$p_{ij} = \frac{q_{ij}}{\sum_{j \neq i} q_{ij}}$$

   and $\mathcal{P}_i$ is the ith row of the matrix P. This results in $H^{\text{any}}_{(i,t)} = \sum_{j \neq i} H^{\text{events}}_{(i,t)}$.

4. Finally, we use the number of households undergoing each transition to compute the number of households in each state at time $t + 1$, per Equation (S1).

We use infection seeding of 10^{-6} and introduce one infected case at the beginning of each year to allow the reintroduction of measles in the population after local extinction during stochastic runs.

Household size	$N = 6$	$N = 5$	$N = 4$
annual	$(0.01, 0.09)$	$(0.01, 0.05)$	$(0, 0.035)$
biennial	$(0.04, 0.18)$	$(0.03, 0.195)$	$(0.04, 0.235)$
triennial	$(0.125, 0.215)$	-	$(0.075, 0.15)$

Table S1: Summary of the bifurcation points for the dynamic transition in the household and age-structured model as the amplitude of seasonality, α is varied.

S4 Comparison of model dynamics under different household size distribution

Comparison of the HH model when the seasonality parameter α is varied reveal differences in dynamical behavior of the model with household sizes. We use the continuation method as described in Section S2 to generate these bifurcation diagrams. The green dots show results from simulations started from the left ($\alpha = 0$) and increasing towards the right. Across all three household sizes considered, we observe commonly associated features of the bifurcation diagrams for measles - annual cycles give way to biennial and triennial cycles as the amplitude of seasonality parameter is increased. As the value of α is increased further (higher than 0.2), chaotic behavior emerges (see Table S1 for bifurcation points). For higher values of α, we also see emergence of large periodic cycles for smaller average household size (compare period 5 outbreaks when $\bar{N}_{\mathbb{C}} = 4$ in Figure S2a with period 6 outbreaks when $\bar{N}_{\mathbb{C}} = 3$ in Figure S2b and period 8 outbreaks when $\bar{N}_{\mathbb{C}} = 2$ in Figure S2c).

The red dots show results from simulations tracing higher-period attractors (triennial/biennial) and moving from right to left. We observe hysteresis loop in the bifurcation diagram for the communities with larger household sizes ($\bar{N}_{\mathbb{C}} = 3$ and 4) that disappears for $\bar{N}_{\mathbb{C}} = 2$.

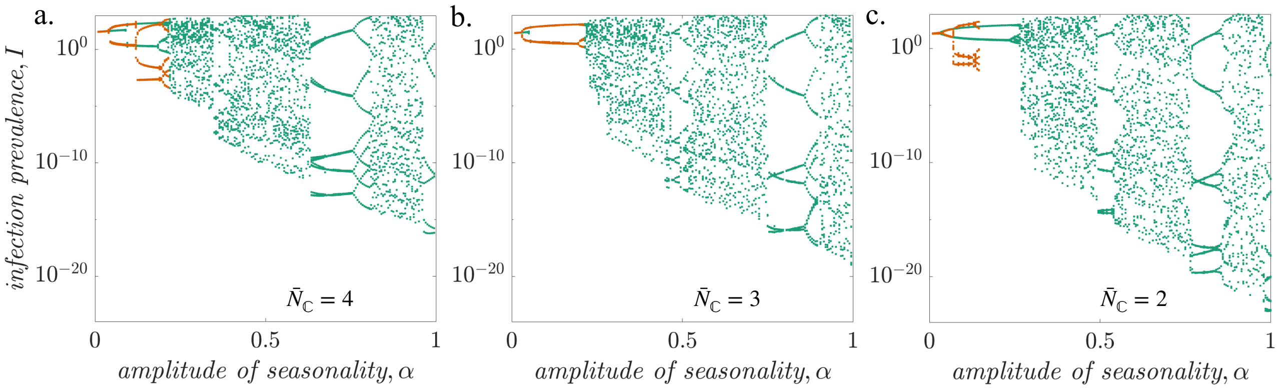

Figure S2: **Comparison of model dynamics with household and age-structured mixing**. Bifurcation diagrams show changes in epidemic dynamics as the amplitude of the sinusoidal forcing term, α is varied for different household sizes: (a) expected number of children $\bar{N}_{\mathbb{C}} = 4$, (b) expected number of children $\bar{N}_{\mathbb{C}} = 3$, and (c) expected number of children $\bar{N}_{\mathbb{C}} = 2$. Each dot represents $\log_{10}$(infectives) sampled annually on 90th day of the year (close to the infection peak) for 20 years after discarding the transient dynamics.

References

[1] Harriet L Mills and Steven Riley. "The spatial resolution of epidemic peaks". *PLoS computational biology* 10.4 (2014), e1003561.

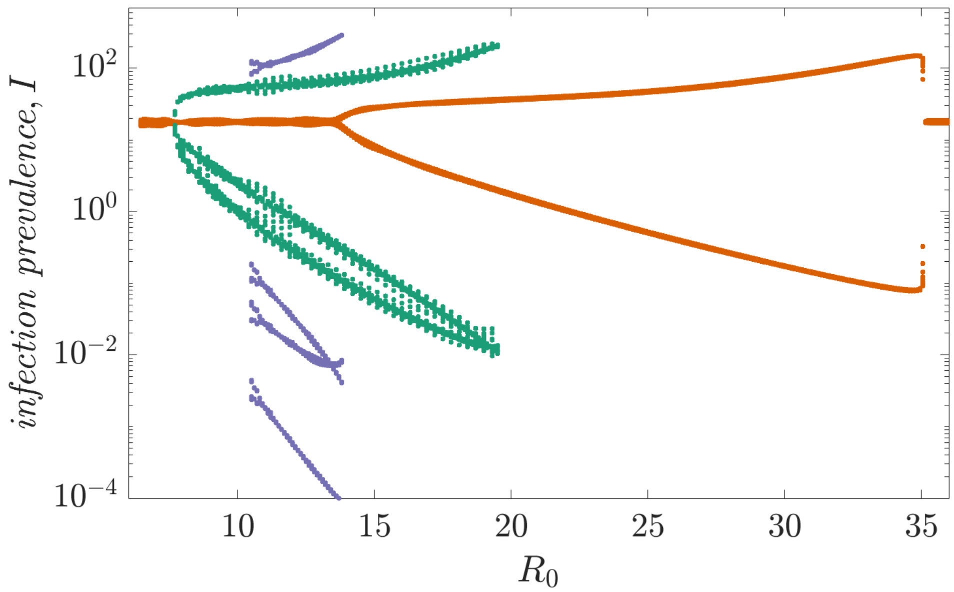

Figure S3: **Bifurcation diagram for wide range of R_0.** Bifurcation diagrams for wider range of R_0 when the expected number of children $\bar{N}_{\mathbb{C}} = 2$.
